# Evaluation of new IPSS-Molecular model and comparison of different prognostic systems in patients with myelodysplastic syndrome

**DOI:** 10.1097/BS9.0000000000000166

**Published:** 2023-07-05

**Authors:** Jiale Ma, Yan Gu, Yanhui Wei, Xuee Wang, Peixuan Wang, Chunhua Song, Zheng Ge

**Affiliations:** a Department of Hematology, Zhongda Hospital, School of Medicine, Southeast University, Institute of Hematology Southeast University, Nanjing 210009, China; b Department of Hematology, Xuzhou Central Hospital, Xuzhou 221009, China; c Hershey Medical Center, Pennsylvania State University Medical College, Hershey, PA, USA; d Division of Hematology, The Ohio State University Wexner Medical Center, the James Cancer Hospital, Columbus, OH, USA

**Keywords:** IPSS-Molecular, myelodysplastic syndrome, prognostic models

## Abstract

A risk-adapted treatment strategy is of crucial importance in patients with myelodysplastic syndromes (MDS). Previous risk prognostic scoring systems did not integrate molecular abnormalities. The new IPSS-Molecular (IPSS-M) model, combing genomic profiling with hematologic and cytogenetic parameters, was recently developed to evaluate the associations with leukemia-free survival (LFS), leukemic transformation, and overall survival (OS). However, it has not yet been widely validated in clinics. This study aims to further validate the prognostic power of IPSS-M based on real-world data and to compare the prognostic value of different scoring systems in patients with MDS. IPSS-M Web calculator was used to calculate a tailored IPSS-M score of the enrolled patient (N = 255), and the risk category was defined correspondingly. We next compared the IPSS-M prognostic power to that of IPSS, IPSS-R, and WPSS. We found that IPSS-M risk classification was statistically significant for 3-year OS and LFS. Compared with other tools, IPSS-M was superior in sensitivity and accuracy for 3-year OS and LFS. The mapping C-index between IPSS-R and IPSS-M categories resulted in improved discrimination across the OS, but not LFS and leukemic transformation. The result of different treatment options indicated that allogeneic hematopoietic stem cell transplantation (allo-HSCT) can result in a better OS than those without allo-HSCT. In conclusion, IPSS-M was a valuable tool for risk stratification compared with other risk prognostic scoring systems. However, more studies should be conducted to explore the appropriate treatment options for different groups stratified by IPSS-M.

## 1. INTRODUCTION

Myelodysplastic syndromes (MDS) are a group of heterogeneous clonal hematopoietic stem cell disorders, characterized by morphologic dysplasia, ineffective hematopoiesis, peripheral blood cytopenia, and high risk of transformation to acute myeloid leukemia (AML).^1^ The heterogeneity of the disease is manifested in the diagnosis, response to treatments, and survival, ranging from indolent conditions with a near-normal life expectancy to forms approaching AML.^2^ A risk-adapted treatment strategy is of crucial importance in patients with MDS.

The International Prognostic Scoring System (IPSS)^3^ based on bone marrow (BM) blasts, cytogenetic abnormalities, and the number of cytopenia was the first published prognostic scoring system written into the National Comprehensive Cancer Network (NCCN) MDS Clinic. Guide.^4^ Limit to the only use for the diagnosis and pretreatment evaluation and the lack of consideration of the severity of cytopenia, the prognostic value of IPSS was questioned. Then, the revised IPSS (IPSS-R) was developed in 2012,^5^ which refined the degree and weight of cytopenia, added more karyotype abnormalities, and assigned higher weights (eg, complex karyotypes) of IPSS, and had been widely used clinically. The WHO-classification-based Prognostic Scoring System (WPSS), which combines WHO type, cytogenetic abnormalities, and anemia, was another prognostic tool for MDS and added the factor of transfusion dependence.^6^ However, all of the previous risk prognostic scoring systems did not integrate molecular abnormalities.

With the rapid development of high-throughput technology like next-generation sequencing (NGS), some somatic mutations have been revealed as significant factors and valuable prognostic biomarkers in MDS.^7–11^ The newly published Molecular International Prognostic Scoring System (IPSS-M), integrating molecular abnormalities with hematologic and cytogenetic data, was recently developed to evaluate the associations with leukemia-free survival (LFS), leukemic transformation, and overall survival (OS).^12^ However, IPSS-M has not yet been widely validated in clinics. This study aims to externally validate this new risk model using real-world patient cohorts, determine its prognostic power in patients with MDS, and explore its utility in guiding therapeutic decisions. We also compared the prognostic value of different scoring systems in patients with MDS.

## 2. METHODS

### 2.1. Patients and inclusion criteria

Data between July 2014 and June 2022 were collected retrospectively from Zhongda Hospital Southeast University (N = 302) and Xuzhou Central Hospital (N = 38). Three hundred forty MDS patients aged 24–95 years old and diagnosed according to World Health Organization (WHO) 2016 criteria^13^ were screened. 255 patients with complete information were finally included in this study. This study was approved by the Independent Ethics Committee for Clinical Research of Zhongda Hospital Southeast University (2023ZDSYLL051-P01) and Xuzhou Central Hospital (XZXY-LK-20230215-003), conducted following the principles of the Declaration of Helsinki.

### 2.2. Clinical, cytogenetic, and molecular markers

Clinical and laboratory data were collected via electronic medical records and databases. We investigated the following clinical characteristics evaluated at diagnosis: age, gender, WHO category, percentage of BM blasts, White blood cell (WBC) count, absolute neutrophil count (ANC), hemoglobin (Hb) level, platelet count (PLT), and cytogenetics.

Unstimulated BM cells were obtained upon initial diagnosis. Cytogenetic analyses were conducted by conventional G-banding technology. Twenty metaphases were analyzed and the karyotypes were described according to the current International System for Human Cytogenetic Nomenclature.^14^ Molecular analyses were conducted by fluorescence in situ hybridization (FISH) and NGS. Genomic DNA was extracted from BM mononuclear cells. Sample integrity was verified using standard NGS criteria (≥50 ng/μL, and OD 260/280:1.8–2.0). We used a custom-targeted NGS approach that combined multiplex PCR-based target enrichment and library generation with ultra-deep high-throughput parallel sequencing using the Ion Proton Platform or MiSeq.^15^ NGS targeted panel analysis was performed in 255 samples where there was available diagnostic material for the risk stratification of IPSS-M (Supplementary methods, http://links.lww.com/BS/A62). IPSS-M was used to restratification with defined 17 main effect genes (ASXL1, CBL, DNMT3A, ETV6, EZH2, FLT3, IDH2, KRAS, MLL^PTD^, NPM1, NRAS, RUNX1, SF3B1^5q^, SF3B1^α^, SRSF2, TP53^multi-hit^, and U2AF1) and 15 residual genes (BCOR, BCORL1, CEBPA, ETNK1, GATA2, GNB1, IDH1, NF1, PHF6, PPM1D, PRPF8, PTPN11, SETBP1, STAG2, and WT1).^12^ The IPSS-M Web calculator (https://mds-risk-model.com) was used to calculate a tailored IPSS-M score for each patient, and the risk category was therefore divided correspondingly.

### 2.3. Treatment methods

Treatment strategies were based on IPSS and IPSS-R, with best supportive care (BSC), immunomodulator therapy (eg, thalidomide, lenalidomide), immunosuppressive therapy (IST, eg, anti-thymocyte globulin or cyclosporine), or hypomethylation agents (HMAs) being chosen for lower-risk patients; and HMAs, HMAs combined with chemotherapy, or allogeneic hematopoietic stem cell transplantation (allo-HSCT) for higher-risk groups.^16^ BSC was defined as blood transfusion, cytokines (eg, EPO, G-CSF, GM-CSF, TPO), and iron chelation. Azacitidine or decitabine was used as HMAs monotherapy. Azacitidine was applied 75 mg/m^2^ daily subcutaneous for 7 days every 28 days. Decitabine was 20 mg/m^2^ daily for 5 days. Combination therapy was typically DAG (daunorubicin, cytarabine, G-CSF), HAG (harringtonine, cytarabine, G-CSF), or CAG (aclacinomycin, cytarabine, G-CSF). In this study, treatment options were divided into five categories, including BSC, immunotherapy (immunomodulator, or IST), HMA monotherapy, HMA combined with chemotherapy, and allo-HSCT.

### 2.4. Survival parameters

Overall survival (OS) was measured from the date of MDS diagnosis to the date of death (uncensored) or last contact (censored). Leukemia-free survival (LFS) was defined as the time from the date of diagnosis to the date of leukemic transformation (uncensored) or death/last contact (censored, whichever occurred first).

### 2.5. Statistical analysis

A Shapiro–Wilk test was used to confirm whether continuous data was a normal distribution. Median values and interquartile range (IQR) were used to describe non-normally distributed data. A Kruskal–Wallis test or analysis of variance t-test was used to compare continuous data. An X^2^ test was used for discrete data. The Kaplan–Meier (K–M) survival curve with log-rank analysis was performed to describe the survival of patients. The reverse K–M method was used to describe the median follow-up time.^17^ The least absolute shrinkage and selection operator (LASSO) regression analyses combined with multivariable Cox regression analyses were used to determine the significant factors that influenced the OS and LFS. Correlative analysis between gene mutations and outcomes was performed on LFS, OS, and AML transformation. Performance and the efficiency of the prognostic models were measured by the Harrell concordance index (C-index) or area under the receiver operating characteristic (ROC) curve (AUC). All statistical analysis and modeling were performed in R version 4.2.3 (The CRAN project, www.r-project.org).

## 3. RESULTS

### 3.1. Patient characteristics and genomic landscape

Data from 255 patients with complete information on IPSS, IPSS-R, WPSS, and IPSS-M scores at diagnosis between July 2014 and June 2022 were evaluated retrospectively from Zhongda Hospital Southeast University (N = 224) and Xuzhou Central Hospital (N = 31) in this study. All patients were identified as MDS with WHO 2016 criteria.^13^ Patients were reclassified according to WHO 2022 criteria in Table 1.^18^ The median age was 66 years (IQR 55–75), the male/female ratio was 1.48:1, and the median follow-up time was 30.6 (95% confidence interval [CI]: 27.0–40.9) months with a median OS of 27.9 (95% CI: 21.3–35.0) months. The clinical characteristics of the MDS patient population were depicted in Table 1.

**Table 1:** Baseline characteristics of MDS patients.

Clinical characteristics	IPSS-M stratification cohort (N = 255)
Median age (IQR), years	66 (55.5, 75.0)
Gender (%)	
Female	103 (40.4)
Male	152 (59.6)
Laboratory parameters	
WBC count, median (IQR), ×10^9^/L	2.6 (1.7, 4.2)
PLT count, median (IQR), ×10^9^/L	57.0 (27.0, 131.5)
Hemoglobin, median (IQR), g/L	71.0 (59.5, 85.0)
ANC, median (IQR), ×10^9^/L	1.3 (0.7, 2.5)
BM blasts, median (IQR), %	0.8 (0.0, 6.4)
2016 WHO Category (%)	
MDS-5q-	3 (1.2)
MDS-SLD	28 (11.0)
MDS-MLD	111 (43.5)
MDS-RS-SLD	6 (2.4)
MDS-RS-MLD	18 (7.1)
MDS-EB-1	44 (17.3)
MDS-EB-2	45 (17.6)
MDS-U	0 (0)
2022 WHO category (%)	
MDS-5q	2 (0.8)
MDS-SF3B1	17 (6.5)
MDS-biTP53	9 (3.4)
MDS-LB	122 (46.7)
MDS-h	16 (6.1)
MDS-IB1	34 (13.0)
MDS-IB2	37 (14.5)
MDS-f	18 (7.0)
MDS category (%)	
Primary MDS	244 (95.7)
(s/t-MDS)	11 (4.3)
IPSS (%)	
Low	36 (14.1)
Intermediate-1	139 (54.5)
Intermediate-2	64 (25.1)
High	16 (6.3)
WPSS (%)	
Very low	14 (5.5)
Low	54 (21.2)
Intermediate	69 (27.1)
High	87 (34.1)
Very high	31 (12.2)
IPSS-R (%)	
Very low	11 (4.3)
Low	69 (27.1)
Intermediate	77 (30.2)
High	51 (20.0)
Very high	47 (18.4)
IPSS-M (%)	
Very low	8 (3.1)
Low	40 (15.7)
Moderate Low	42 (16.5)
Moderate high	51 (20.0)
High	52 (20.4)
Very high	62 (24.3)
Karyotype, n (%)	
del(5q)	
No	218 (85.5)
Yes	37 (14.5)
Single del(5q)	10 (3.9)
del(7q)/−7	
No	227 (89.0)
Yes	28 (11.0)
Single del(7q)/−7	9 (3.5)
del(17p)/−17	
No	245 (96.1)
Yes	10 (3.9)
Single del(17p)/−17	2 (0.8)
Complex (>3 abnormalities)	
No	225 (88.2)
Yes	30 (11.8)
Cytogenetics category (%)	
Very good	3 (1.2)
Good	163 (63.9)
Intermediate	45 (17.6)
Poor	16 (6.3)
Very poor	28 (11.0)
Treatment (%)	
BSC	114 (44.7)
Immunotherapy	21 (8.2)
HMA monotherapy	125 (49.0)
HMA + chemotherapy	22 (8.6)
Allo-HSCT	17 (6.7)

Allo-HSCT = allogeneic hematopoietic stem cell transplantation, ANC = absolute neutrophil count, BM = bone marrow, BSC = best supportive care, HMAs = hypomethylation agents, IPSS = International Prognostic Scoring System, IPSS-M = Molecular International Prognostic Scoring System, IPSS-R = Revised International Prognostic Scoring System, IQR = interquartile range, MDS = myelodysplastic syndromes/neoplasms, PLT = platelet, s/t-MDS = secondary/therapy-related MDS, WBC = white blood cell count, WPSS = World Health Organization-classification-based Prognostic Scoring System.

**Figure 1.**
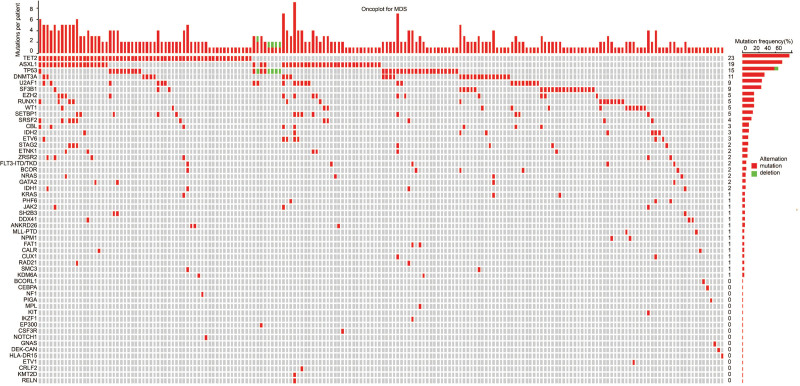
The frequency of mutated genes in 255 MDS samples is shown. Colors represent the type of lesions. A histogram of the total number of oncogenic alterations (gene mutations) per patient (top) and gene mutation frequency (right side) is also shown. MDS = myelodysplastic syndromes/neoplasms.

We mapped at least one oncogenic gene alteration in patients with complete information for gene mutations and found that TET2 (23%), ASXL1 (19%), TP53 (15%), DNMT3A (10%), U2AF1 (9%), SF3B1 (9%), EZH2 (9%), RUNX1 (5%), WT1 (5%), and SETBP1 (5%) are the top 10 genes with the highest mutation frequency (Fig. 1). To identify all independent gene mutation factors for OS and LFS, we next performed LASSO regression analysis combined with multivariable Cox regression analysis in the MDS cohort. LASSO regression analysis demonstrated that mutations in DNMT3A, SRSF2, PPM1D, CALR, and KDM6A were the prognostic factors for OS, but not for LFS (Figure S1, http://links.lww.com/BS/A62). Next, in multivariable analysis, we confirmed that DNMT3A, SRSF2, PPM1D, and KDM6A were significant predictors for OS (*P* < .05) and associated with adverse prognoses (Table S3, http://links.lww.com/BS/A62). It was worth noting that CALR mutations were detected in three patients, in which, two (MDS-f and MDS-LB subtype, respectively) were type I mutations,^19^ and the other one (MDS-f) was type II mutation.^19^ All three patients were transformed from primary myelofibrosis with ≥grade 2 myelofibrosis. Whether CALR mutations were hotspot mutations in MDS-f and the potential pathogenesis need to be further evaluated.

### 3.2. Validation of the prognostic effect of IPSS-M in the MDS population

**Figure 2.**
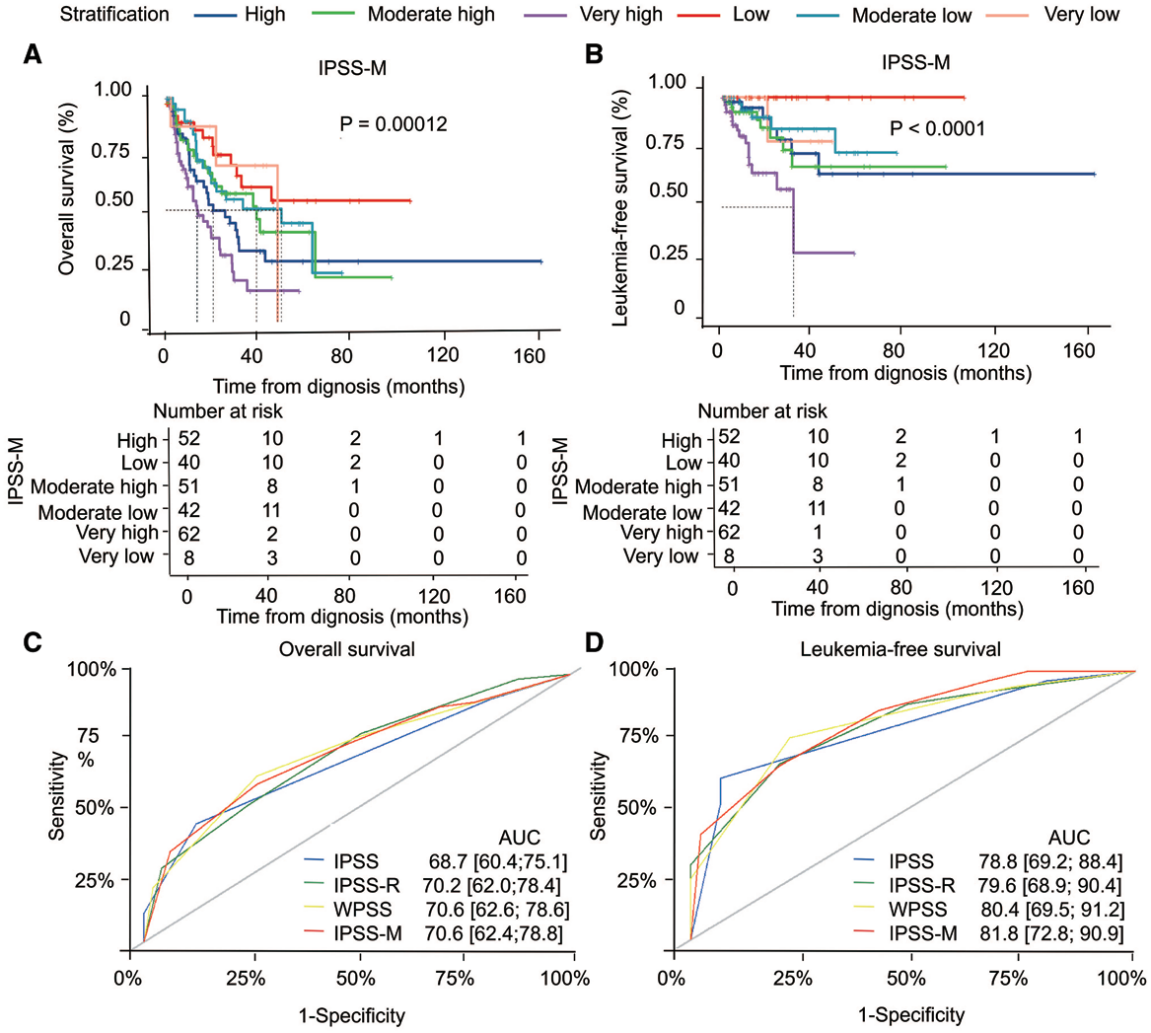
Application of newly developed IPSS-M risk classification. (A) Kaplan–Meier probability estimates of OS stratified by IPSS-M. (B) Kaplan–Meier probability estimates of LFS stratified by IPSS-M. P values are from the log-rank test. The receiver operating characteristic curves of (C) OS and (D) LFS of different risk stratifications. The AUC value represents the area under the ROC curve.AUC = receiver operating characteristic curve, IPSS = International Prognostic Scoring System, IPSS-M = Molecular International Prognostic Scoring System, IPSS-R = Revised International Prognostic Scoring System, LFS = leukemia-free survival, OS = overall survival, ROC = receiver operating characteristic, WPSS = World Health Organization-classification-based Prognostic Scoring System.

We first calculated individual IPSS-M score parameters by the IPSS-M Web calculator to evaluate the prognostic effect on the enrolled MDS patients (N = 255). By stratifying patients by IPSS-M score, the median OS was 48.1, 45.4, 33.03, 37.6, 20.3, and 13.3 months for the very low, low, moderate low, moderate high, high, and very high groups, respectively. Similar to the other three prognostic systems, IPSS-M was statistically significant for OS (*P* = .00012, Figs. 2A, S2a–c, http://links.lww.com/BS/A62) and LFS (*P* < .0001, Figs. 2B, S2d–f, http://links.lww.com/BS/A62). The IPSS-M risk categories demonstrated prognostic separation across all endpoints, especially in high/very high and low/very low-risk groups (*P* = .00012, Fig. 2A), but the survival curves still intersect between moderate high and moderate low groups, indicating that the discrimination between these two groups was not obvious. Although median LFS were not reached for all groups, the LFS in higher-risk groups (HR, including moderate high, high, and very high groups) were shorter than lower-risk groups (LR, including moderate low, low, and very low groups) (*P* = .00065, Figure S2g, http://links.lww.com/BS/A62).

### 3.3. Comparison of the prognostic power of different prognostic systems

To assess the quality and value of the IPSS-M model in patients with MDS (N = 255), we compared the AUCs of IPSS-M with IPSS, IPSS-R, and WPSS. Stratified by IPSS, IPSS-R, WPSS, or IPSS-M, the AUC for 3-year OS was 0.687 (95% CI: 0.604–0.751), 0.702 (0.62–0.784), 0.706 (0.626–0.786), 0.706 (0.624–0.788); and 3-year LFS was 0.788 (0.692–0.884), 0.796 (0.689–0.904), 0.804 (0.695–0.912), 0.818 (0.728–0.909), respectively (Fig. 2C and D). Since the higher AUC is associated with better diagnostic performance of the model,^20^ the present results indicated that IPSS-M was more superior in sensitivity and accuracy for both OS and LFS compared with the previous risk stratification tools.

### 3.4. Restratification of patients from IPSS-R to IPSS-M

To compare IPSS-R and IPSS-M patients’ distribution, we analyzed the restratification of MDS patients (N = 255) (Table 2). Restratified by IPSS-M, a total of 114 patients (44.7%) were reclassified (Table 2). More than half of patients classified as IPSS-R low shifted (39/69, 56.5%); 31 of 69 (44.9%) were upstaged to IPSS-M moderate low and moderate high groups (Table S4, http://links.lww.com/BS/A62). This demonstrated that reclassification occurred in almost half of the patients when integrating molecular abnormalities into clinical parameters. To further explore the implications of this restratification, we analyzed the outcomes between IPSS-M categories within each IPSS-R stratum. There were no statistically differences in outcomes between IPSS-M in each IPSS-R category (*P* > .05, Figure S3, http://links.lww.com/BS/A62).

**Table 2:** Distribution of MDS patients categorized by IPSS-M and IPSS-R.

IPSS-R (n, %)	IPSS-M (n, %)						
	Very high	High	Moderate high	Moderate low	Low	Very low	Total
Very high	36 (14.12)	8 (3.14)	3 (1.18)	0	0	0	47 (18.43)
High	20 (7.84)	21 (8.24)	10 (3.92)	0	0	0	51 (20)
Intermediate	5 (1.96)	18 (7.06)	25 (9.80)	23 (9.02)	6 (2.35)	0	77 (30.20)
Low	1 (0.39)	5 (1.96)	13 (5.10)	18 (7.06)	30 (11.76)	2 (0.78)	69 (27.06)
Very low	0	0	0	1 (0.39)	4 (1.57)	6 (2.35)	11 (4.31)
Total	62 (24.31)	52 (20.39)	51 (20)	42 (16.47)	40 (15.69)	8 (3.14)	255 (100)

IPSS-M = Molecular International Prognostic Scoring System, IPSS-R = Revised International Prognostic Scoring System, MDS = myelodysplastic syndromes/neoplasms.

**Figure 3.**
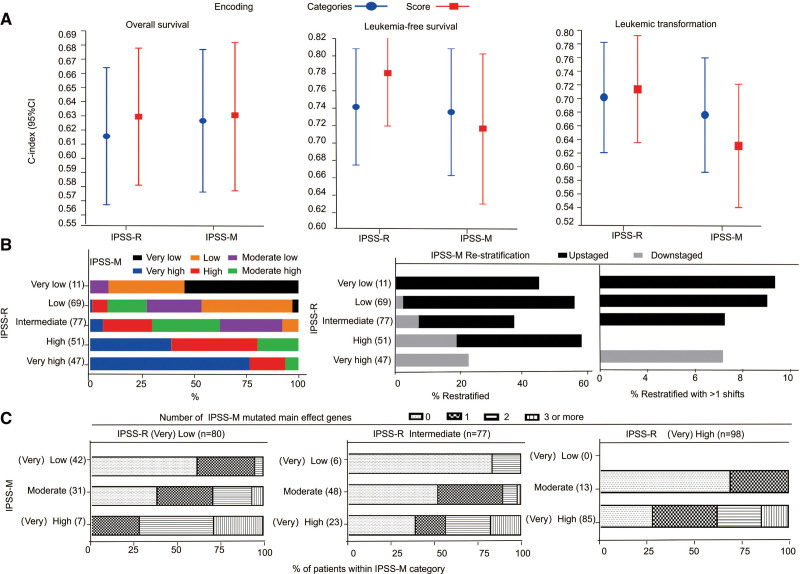
Comparison of the IPSS-R and IPSS-M. (A) Model discrimination as measured by the C-index obtained with the IPSS-R or the IPSS-M across the three endpoints. (B) Stacked bar plots show the restratification of IPSS-R to IPSS-M for 255 patients where both scores could be calculated. Each row corresponds to one IPSS-R category, and colors represent the IPSS-M categories. (C) The association between the numbers of mutated IPSS-M main effect genes and restratification. IPSS-M = Molecular International Prognostic Scoring System, IPSS-R = Revised International Prognostic Scoring System.

Compared the C-indexes of scores and categories for IPSS-R and IPSS-M, we found that IPSS-M categories resulted in the improved discrimination across the OS but not LFS, or leukemic transformation. The C-index was 0.63 (95% CI: 0.58–0.68) versus 0.62 (95% CI: 0.57–0.67) for OS, 0.735 (95% CI: 0.66–0.81) versus 0.741 (95% CI: 0.67–0.81) for LFS, and 0.67 (95% CI: 0.60–0.76) versus 0.71 (95% CI: 0.63–0.79) for leukemic transformation, respectively (Fig. 3A). Next, we compared the C-indexes of categories for IPSS-R and IPSS-M among the restratification patients (N = 114), the result indicated that IPSS-M improved discrimination across all endpoints compared to IPSS-R, with the C-indexes of 0.653 versus 0.615 for OS, 0.797 versus 0.788 for LFS, and 0.766 versus 0.742 for leukemic transformation, respectively (Table 3).

**Table 3:** C-indexes of IPSS-R and IPSS-M categories in restratification patients.

Outcomes	Stratification systems			
	IPSS-R		IPSS-M	
	C-index	Hazard ratio (95% CI)	C-index	Hazard ratio (95% CI)
Overall survival	0.615	0.537–0.693	0.653	0.577–0.729
Leukemia-free survival	0.788	0.704–0.829	0.797	0.693–0.901
Leukemic transformation	0.742	0.613–0.871	0.766	0.669–0.863

IPSS-M = Molecular International Prognostic Scoring System, IPSS-R = Revised International Prognostic Scoring System, MDS = myelodysplastic syndromes/neoplasms.

IPSS-M resulted in the restratification of 44.7% (114/255) in the study cohort (Fig. 3B). Among patients restratified (N = 114), 29 of 114 (25.4%) were downstaged, while 85 of 114 (74.6%) were upstaged (Fig. 3B, Table S5, http://links.lww.com/BS/A62). Simplified risk categories were obtained by merging the very low/low categories into low, and very high/high categories into high for both IPSS-R and IPSS-M. Among patients of simplified risk categories, 81 of 255 (31.8%) had one main effect gene in IPSS-M, whereas 64 of 255 (25.1%) had two or more, the distribution of the number of mutated IPSS-M main effect genes in patient’s risk categories was shown in Figure 3C and Table S6, http://links.lww.com/BS/A62. In detail, among restratified patients by simplified risk categories, 20 of 80 (25%) had one mutated IPSS-M main effect gene, whereas 25 of 80 (31.3%) had two or more mutated genes. Thus, the main effect genes in IPSS-M played key roles in the restratification of MDS patients, and patient restratification was not a single gene effect, but the cumulative contribution of the prognostic mutations for each patient.

### 3.5. Treatment effects on different groups stratified by IPSS-M

**Figure 4.**
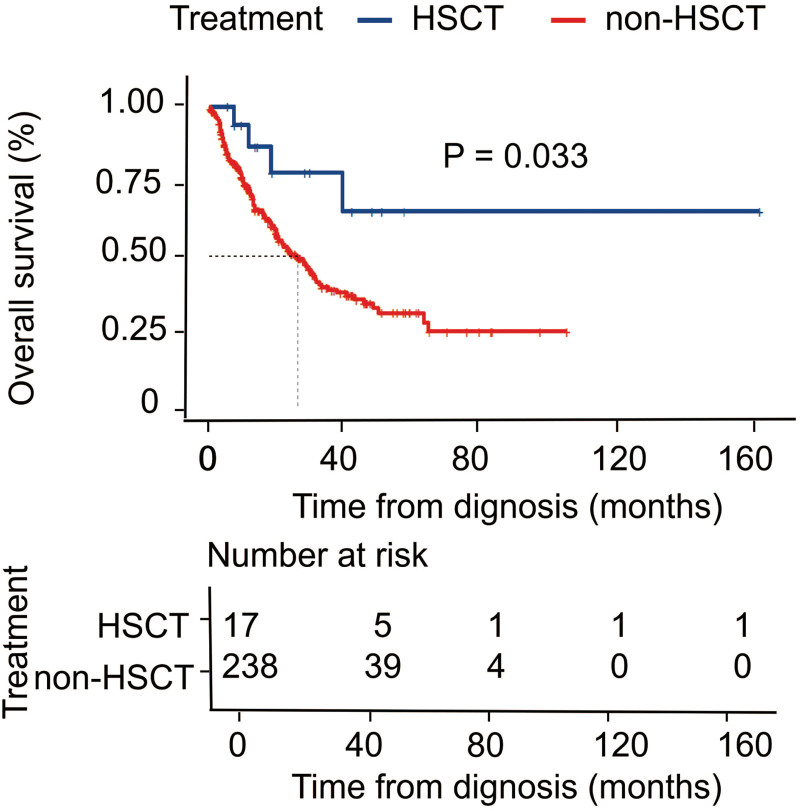
Kaplan–Meier probability estimates of OS for HSCT vs non-HSCT in patients with MDS. HSCT = hematopoietic stem cell transplantation, MDS = myelodysplastic syndromes/neoplasms, OS = overall survival.

**Figure 5.**
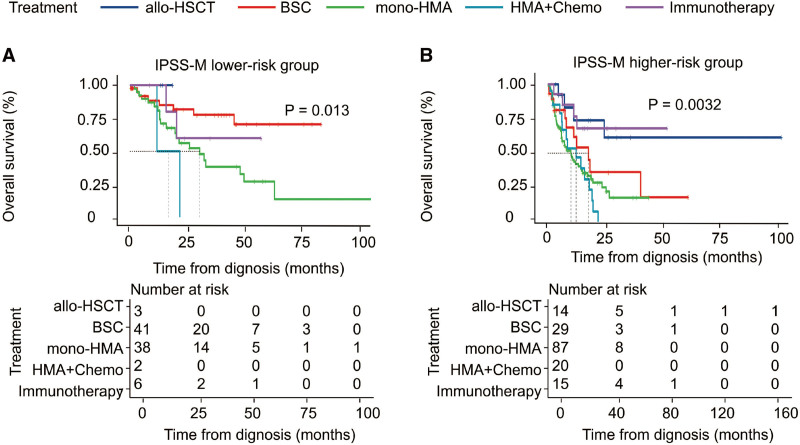
Kaplan–Meier probability estimates of OS for patients who were treated with different therapy in the IPSS-M lower- and higher-risk categories. The *P* value is from the log-rank test. IPSS-M = Molecular International Prognostic Scoring System, OS = overall survival.

To further address the therapeutic utility of the IPSS-M, we assessed the outcome among each IPSS-M group based on disease-modifying therapy with BSC, immunotherapy (including immunomodulator and IST), HMA monotherapy, HMA combined with chemotherapy, and allo-HSCT. Among the whole cohort, patients who underwent HSCT had a better OS than those non-HSCT (*P* = .033, Figures S4 and S5, http://links.lww.com/BS/A62), especially in IPSS-M very high-risk group (*P* = .029, Fig. 4). For patients in the IPSS-M LR group, there was a significant survival benefit for patients treated with BSC in our study (*P* = .013, Fig. 5). For patients in the IPSS-M HR group, there was a significant survival benefit for those patients who underwent allo-HSCT compared to those who were treated with other treatment strategies (*P* = .0032, Fig. 5).

We next analyzed the K–M survival curves of different groups stratified by IPSS-M and IPSS-R who received the same therapy (Figure S6, http://links.lww.com/BS/A62). The result showed that there was significant for these two scoring systems in patients treated with HMAs (*P* = .024 in IPSS-M, and *P* = .011 in IPSS-R). Additionally, for patients treated with HMAs, IPSS-M was superior in both sensitivity and applicability than IPSS-R in OS and LFS, with a C-index of 0.615 versus 0.598, and 0.673 versus 0.654, respectively (Table S7, http://links.lww.com/BS/A62). However, in patients treated with BSC, immunotherapy, or allo-HSCT, IPSS-M was superior in both sensitivity and applicability than IPSS-R in OS but not LFS (Table S7, http://links.lww.com/BS/A62). These results indicated that IPSS-M was superior to IPSS-R for predicting OS, regardless of the treatment. IPSS-M was more applicable for predicting OS and LFS in those who received HMAs treatment with relatively higher C-index values than IPSS-R.

## 4. DISCUSSION

MDS is a group of highly heterogeneous myeloid clonal diseases originating from hematopoietic stem cells. Due to the highly heterogeneous natural history and prognosis, treatment for MDS should be individualized. Currently, IPSS-R is the most widely adopted tool for risk stratification, prognostic assessment, and individual treatment decisions making in newly diagnosed MDS patients.^21^ Previous studies have demonstrated the effects of gene mutations in the outcomes of patients with MDS,^7,9,11,22^ and some have been intended to establish personalized prediction models based on clinical and genomic data in MDS.^9,11^ However, none of them have been integrated into a comprehensive prognostic system and uniformly applied in clinical practice. In 2022, the newly published IPSS-M model, which consists of blood counts, marrow blasts, the five IPSS-R cytogenetic categories, 16 main effect genes, and 15 residual genes, combining genomic data with hematologic and cytogenetic parameters in the IPSS-R system, bringing a major change to the risk classification of MDS.^12^ The IPSS-M model was developed and validated in data from 3711 patients and was the largest prognostic model with the largest sample size to date.^12^

In this study, we evaluated the prognostic value of IPSS-M within two centers’ MDS population cohorts. We confirmed that IPSS-M risk classification was statistically significant for OS and LFS. However, different from the Bernard study, IPSS-M did not result in a significant improvement for discriminating moderate risk groups, with the survival curves intersecting between moderate high and moderate low groups on K–M OS curves.^12^ This might be related to the sample size limitations and data bias. When comparing IPSS-M with IPSS-R, high concordance for risk categories was observed, suggesting that both systems well represent the prognostic risk of MDS patients. Discrepancies are mainly involved in the number of main effect genes.

Due to the highly heterogeneous, the outcome of MDS patients ranges from conditions with a near-normal life expectancy to variants rapidly progressing to AML.^23^ The clinical heterogeneity likely reflects different driver mutations responsible for the clonal proliferation of myelodysplastic stem cells.^24^ Mutations in genes implicated in RNA splicing, DNA modification, chromatin regulation, and cell signaling have been identified by the sequencing of MDS genomes.^25^ In IPSS-M, the main effect genes and residual genes have the adverse effects of similar amplitude with consistent directions across endpoints.^12^ In our study, we verified that the main effect genes such as DNMT3A and SRSF2, and residual gene PPM1D were significantly associated with poorer prognosis. Meanwhile, the OS and LFS deteriorated as the number of driver mutations increased. CALR mutations were detected in three of 255 of patients with ≥ grade 2 myelofibrosis, in which, two patients were defined as MDS-f subtype according to WHO 2022 classification. A larger scale of clinical studies should be performed to confirm the frequency and the potential pathogenesis involving CALR mutations in the transformation from MPN to MDS (especially MDS-f). Additionally, we also found that mutation in KDM6A was related to the poor prognosis. KDM6A is a histone demethylase and plays a critical role in hematopoietic differentiation and differentiation-specific gene expression programs.^25,26^ Whether KDM6A acts as a tumor suppressor or oncogene is likely dependent on cellular context.^27,28^ Conditional knockout KDM6A induced myelodysplasia and splenic erythropoiesis in female mice, suggesting a tumor suppressor function of wildtype KDM6A.^29^ While KDM6A was a pro-oncogenic cofactor essential for leukemia maintenance in TAL1-positive T-ALL, suggesting an oncogenic function.^30^ Therefore, analysis of MDS-specific mutations of KDM6A is required to clarify its pathophysiologic function, and more gene mutation files should be included to further identify the prognostic factors.

When compared with IPSS-R, IPSS-M demonstrated higher sensitivity and accuracy for OS. Despite the differences in the conception between IPSS-M and IPSS-R, when comparing the two systems, a high concordance was observed. Discrepancies in patient risk classification mainly involved patients with gene mutations. Therefore, for patients who are ineligible for NGS, IPSS-R can still be an option stratification tool for clinical decision-making.

The IPSS-M system did not indicate the appropriate treatment options for different groups of MDS patients.^12^ To further explore treatment effects on different groups stratified by IPSS-M, we compared the OS for patients who received HMAs monotherapy or combined with chemotherapy, BSC or immunotherapy, and allo-HSCT. The benefit of allo-HSCT was observed in most risk patients, especially in higher-risk groups. This indicated that for patients with higher IPSS-M risk groups, allo-HSCT will be the better choice. Interestingly, the survival benefit was not observed in lower- and intermediate-risk groups for patients with HMAs therapy. Since our study was retrospective and cannot account for the decision variables of the treating physicians, patients included in this study were older age, had poorer physical status, and had a higher incidence of infectious complications. All these factors resulted in poor OS in HMAs treatment groups. Therefore, treatment decisions should be individualized by considering other risk features such as age, presence of BM fibrosis, serum lactate dehydrogenase level, serum ferritin, or incorporation of molecular data in these settings.^23,31,32^ Additionally, more real-world studies are needed to validate the prognostic value of the IPSS-M using a large external cohort of actively treated MDS patients, including patients who received disease-modifying therapy.

The limitations of our study should be noted. In the present study, the IPSS-M-defined genes were not fully covered in the screening gene panel due to the retrospective nature of the data, although all of the 16 main effect genes for IPSS-M were covered. Sauta et al analyzed the accuracy of IPSS-M prediction when molecular information was missed and demonstrated that information on the mutational status of a set of 15 genes (ASXL1, CBL, DNMT3A, ETV6, EZH2, FLT3, IDH2, MLL^PTD^, NPM1, NRAS, RUNX1, SF3B1, SRSF2, TP53^multihit^, and U2AF1) was required to have an accuracy of IPSS-M prediction of 80% and 70% in the GenoMed4all (n = 2876) and IWG-PM cohorts (n = 2957), respectively.^33^ In this study, all the reported 15 required genes are included, which strengthens the accuracy of IPSS-M prediction.

IPSS-M provides us with a robust tool for conducting risk-based clinical trials and precision medicine-based treatment options. Despite the apparent refinements, the IPSS-M does have limitations. First, the score is relatively complicated, and its general adoption in the community setting may pose challenges. Second, molecular testing is not yet routine globally because of cost, infrastructure, and reimbursement considerations, which will limit the applicability of IPSS-M. A more streamlined selection of the highest-priority molecular markers would help address these limitations. Thirdly, the appropriate treatment options for MDS patients in different groups were not pointed out and the prognostic differences among different treatment regimens were not indicated. Therefore, large series of cases are needed to validate the clinical application of the new IPSS-M and large scales of clinical trials should be conducted to explore the appropriate treatment options for MDS patients in different groups stratified by IPSS-M.

## 5. CONCLUSION

In conclusion, IPSS-M was a valuable tool for risk stratification. It had an improved prognostic power for OS compared to the IPSS, WPSS, and IPSS-R. More real-world studies should be conducted to provide the information for risk-adapted strategies in MDS.

## ACKNOWLEDGMENT

This work is supported in part by Medical research key projects-Jiangsu Commission of Health (ZD2021003).

## AUTHOR CONTRIBUTIONS

J.M., Y.G., Y.W., X.W., and P.W. were involved in the collection and assembly of the data. J.M. performed data analysis and wrote the manuscript. Z.G. and C.S. designed the study and edited the manuscript. All authors have read and approved the manuscript.

## Supplementary Material


