# Supplementary material for: Evaluation of new IPSS-Molecular model and comparison of different prognostic systems in patients with myelodysplastic syndrome
Source: Blood Sci. 2023 Jul 5;5(3):187–95. doi: 10.1097/BS9.0000000000000166 (PMC10400062; doi:10.1097/BS9.0000000000000166)

Supplementary Appendix

Evaluation of new IPSS-Molecular model and comparison of different prognostic systems in patients with myelodysplastic syndrome

Jiale Ma[1,2#], Yan Gu[1#], Yanhui Wei[1], Xuee Wang[1], Peixuan Wang[1], Chunhua Song[3,4], Zheng Ge[1*]

[1] Department of Hematology, Zhongda Hospital, School of Medicine, Southeast University, Institute of Hematology Southeast University, Nanjing 210009, China.
[2] Department of Hematology, Xuzhou Central Hospital, Xuzhou 221009, China.
[3] Hershey Medical Center, Pennsylvania State University Medical College, Hershey, USA.
[4] Division of Hematology, The Ohio State University Wexner Medical Center, the James Cancer Hospital, Columbus, USA.

#These authors contributed equally to the work.

*Correspondence to:
Zheng Ge, M.D., Ph.D.
Department of Hematology
Zhongda Hospital, School of Medicine, Southeast University
Institute of Hematology Southeast University
No. 87, Dingjiaqiao, Nanjing 210009, China,
Telephone: 86-25-83262468
FAX: 86-25-83262471
E-mail: zhengge@seu.edu.cn
ORCID: orcid. org/0000-0001-8028-1612

Table of contents

1. Supplementary Methods 3
1.1 Targeted Genotyping Panel...3
Table S1 Gene panel for MDS or MDS/MPN...3
Table S2 Gene panel for myeloid malignancies and acute leukemia............3
2. Supplementary Results..4
2.1 Supplementary Tables...4
Table S3 Results of LASSO and COX analysis..4
Table S4 Restratification matrix of the number of patients classified in each of the 5 IPSS-R category and each of the 6 IPSS-M category...............................4
Table S5 Restratification of numbers of patients classified of each IPSS-R group to IPSS-M...5
Table S6 The association between the numbers of mutated IPSS-M main effect genes and restratification.. 6
Table S7 C-index values for IPSS-R and IPSS-M in patients with different treatment strategies...7
2.2 Supplementary Figures 7
Figure S1 The process of selecting variables by LASSO regression analysis...8
Figure S2 Kaplan-Meier probability estimates of OS and LFS for patients stratified by different prognostic systems..9
Figure S3 Kaplan-Meier probability estimates of survival outcome per IPSS-M category within each IPSS-R category..10
Figure S4 Kaplan-Meier probability estimates of OS for patients who were treated with different therapy per IPSS-M category..11
Figure S5 Kaplan-Meier probability estimates of OS for different treatment options in patients with MDS...12
Figure S6 Kaplan-Meier probability estimates of OS in different groups stratified by IPSS-M and IPSS-R who received the same therapy.......................13

1. Supplementary Methods

1.1 Targeted Genotyping Panel

According to the recommends of 2008, 2017 and 2022 revision to the World Health Organization (WHO) classification of myeloid neoplasms and acute leukemia, various expert consensuses, authoritative literature, research reports in recent years, a bait-capture gene panel consisting of 35 or 85 genes known to be recurrently mutated in MDS, MDS/MPN or myeloid malignancies and acute leukemia were selected for detection. Putative genes were defined as MDS, MDS/MPN, or myeloid malignancies and acute leukemia-related genes, which were detected by Deoxyribonucleic acid sequencing using the Nextseq550 system, a probe capture library construction method, with an average sequencing depth of more than 1000×. The putative genes were screened by dbSNP, 1000G, EXAC, COSMIC, HGMD, GnomAD, and other databases built by our laboratory. SIFT and Polyphen2 databases were used to predict and annotate the structure of amino acid point mutations, and the putative genes were mutations with less than 1% mutation frequency in each database, and benign mutation sites were excluded. The mean mutation detection sensitivity of Gene panels in this paper was 3%, and the mutation detection sensitivity of FLT3-ITD was 1% by PCR capillary electrophoresis.

Table S1 Gene panel for MDS or MDS/MPN

ASXL1	DNMT3A	GATA1	JAK3	NRAS	SETBP1	TET2
BCOR	ETNK1	GATA2	KRAS	PHF6	SF3B1	TP53
CALR	ETV6	IDH1	MPL	PPM1D	SH2B3	U2AF1
CBL	EZH2	IDH2	NF1	PTPN11	SRSF2	WT1
DDX41	FLT3	JAK2	NPM1	RUNX1	STAG2	ZRSR2

Table S2 Gene panel for myeloid malignancies and acute leukemia

ABL1	CEBPA	ETNK1	IL7R	NF1	RAD21	SOS1
ANKRD26	CEBPE	ETV6	JAK1	NOTCH1	RAF1	SRP72
ARID5B	CRLF2	EZH2	JAK2	NPM1	RARA	SRSF2
ASXL1	CSF3R	FAT1	JAK3	NRAS	RB1	STAG2
ASXL2	CSMD1	FBXW7	KANSL1	PAX5	RUNX1	TET2
BCOR	CTCF	FLT3	KDM6A	PDGFRA	SETBP1	TP53
BCORL1	DDX41	GATA1	KIT	PDGFRB	SETD2	U2AF1
BRAF	DHX15	GATA2	KMT2A	PHF6	SF3B1	WT1
CALR	DNMT3A	GATA3	KRAS	PIGA	SH2B3	ZBTB7A
CBL	ECT2L	IDH1	MECOM	PPM1D	SMC1A	ZRSR2
CDKN2A	EED	IDH2	MPL	PTEN	SMC2	
CDKN2B	EP300	IKZF1	MYC	PTPN11	SMC3	

2. Supplementary Results

2.1 Supplementary Tables

Table S3 Results of LASSO+COX analysis

Genes	Hazard ratio (95%CI)	P-value
DNMT3A	1.88(1.1-3.21)	0.020
SRSF2	3.81(1.82-8.00)	0.000
PPM1D	6.92(1.17-28.44)	0.007
CALR	1.921e-07(0-Inf)	0.990
KDM6A	9.87(2.34-40.9)	0.002

Abbreviations: CI, confidence interval

Table S4 Restratification matrix of the number of patients classified in each of the 5 IPSS-R category (row) and each of the 6 IPSS-M category (column)

Category		IPSS-M						
		Very high	High	Moderate high	Moderate Low	Low	Very low	Total
IPSS-R	Very high	36 (76.6%)	8 (17%)	3 (6.4%)	0	0	0	47
	High	20 (39.2%)	21 (41.2%)	10 (19.6%)	0	0	0	51
	Intermediate	5 (6.5%)	18 (23.4%)	25 (32.5%）	23 (29.8%)	6 (7.8%)	0	77
	Low	1 (1.4%)	5 (7.3%)	13 (18.8%)	18 (26.1%)	30 (43.5%)	2 (2.9%)	69
	Very low	0	0	0	1 (9.1%)	4 (36.4%)	6 (54.5%)	11
	Total	62	52	51	42	40	8	255

Table S5 Restratification of numbers of patients classified of each IPSS-R group to IPSS-M

Category		IPSS-M (n%) re-stratified (n=114)		IPSS-M (n%) re-stratified with >1 shifts (n=15)	
		Downstaged (n=29)	Upstaged (n=85)	Downstaged (n=3)	Upstaged (n=12)
IPSS-R (n%)	Very high (n=47)	11(23.4)	0	3(6.4)	0
	High (n=51)	10(19.6)	20(39.2)	0	0
	Intermediate (n=77)	6(7.8)	23(29.9)	0	5(6.5)
	Low (n=69)	2(2.9)	37(53.6)	0	6(8.7)
	Very low (n=11)	0	5(45.5)	0	1(9.1）

Table S6 The association between the numbers of mutated IPSS-M main effect genes and restratification

Category	IPSS-R(Very) Low (n=80) (n%)			
**IPSS-M Numbers of mutated main effect genes**	0	1	2	>=3
(Very) High(n=7)	0	2(28.5)	3(43)	2(28.5)
Moderate (n=31)	12(38.7)	10(32.2)	7(22.6)	2(6.5)
（Very) Low(n=42)	26(61.9)	14(33.3)	2(4.8)	0
**Category**	**IPSS-R Intermediate (n=77) (n%)**			
**IPSS-M Numbers of mutated main effect genes**	0	1	2	>=3
(Very) High (n=23)	9(39.1)	4(17.4)	6(26.1)	4(17.4)
Moderate(n=48)	25(52.1)	18(37.5)	4(8.3)	1(2.1)
(Very) Low (6)	5(83.4)	0	1(16.6)	0
**Category**	**IPSS-R (Very) High (n=98) (n%)**			
**IPSS-M Numbers of mutated main effect genes**	0	1	2	>=3
(Very) High (n=85)	24(28.2)	29(34.1)	20(23.5)	12(14.1)
Moderate(n=13)	9(69.3)	4(30.7)	0	0
(Very) Low	0	0	0	0

Table S7 C-index values for IPSS-R and IPSS-M in patients with different treatment strategies

**Outcomes**	**Stratification systems**	**Best supportive care or immunotherapy**		**Hypomethylation agents**		**Allo-HSCT**	
		C-index	Hazard ratio(95%CI)	C-index	Hazard ratio(95%CI)	C-index	Hazard ratio(95%CI)
Overall survival	IPSS-M	0.624	0.528-0.719	0.615	0.554-0.676	0.681	0.485-0.877
	IPSS-R	0.613	0.525-0.701	0.598	0.537-0.659	0.569	0.214-0.924
Leukemia-free survival	IPSS-M	0.818	0.646-0.990	0.673	0.577-0.769	0.679	0.430-0.928
	IPSS-R	0.858	0.748-0.968	0.654	0.558-0.750	0.964	0.891-1.036

2.2 Supplementary Figures

Figure S1 The process of selecting variables by LASSO regression analysis. (a) and (b) for OS, (c) and (d) for LFS. OS, overall survival; LFS, leukemia-free survival;

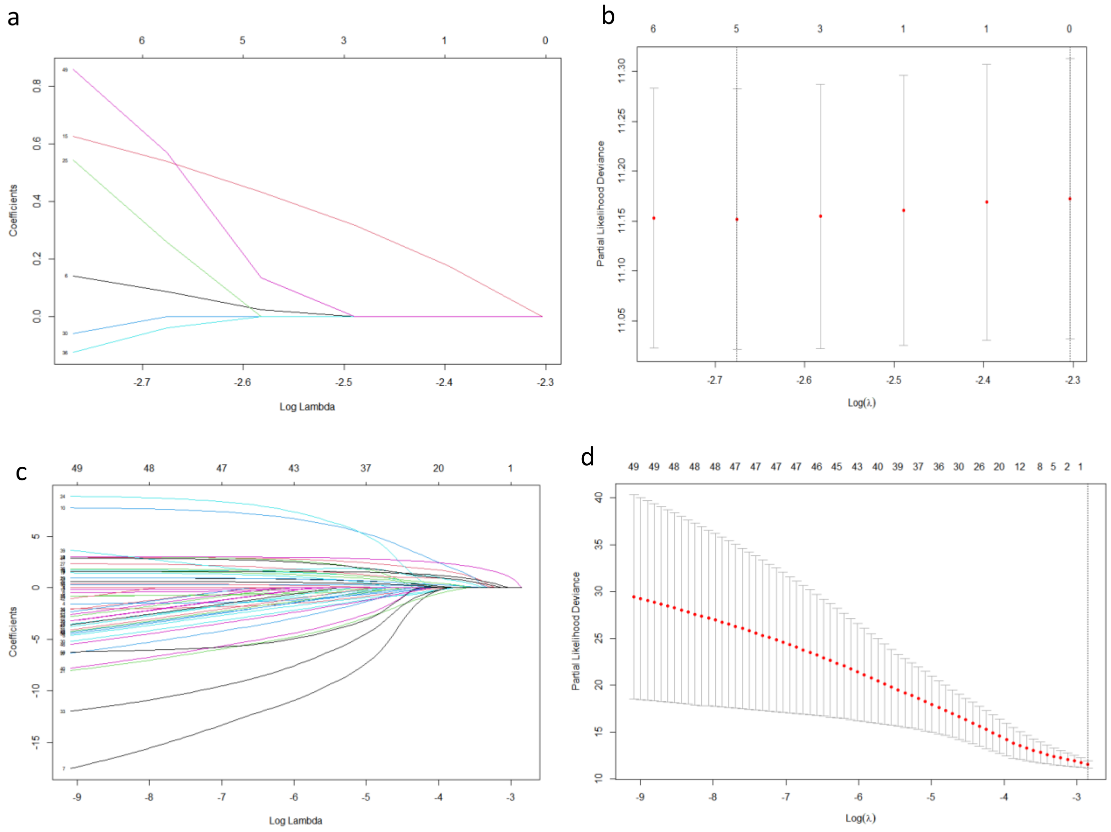

Figure S2 Kaplan-Meier probability estimates of OS and LFS for patients stratified by different prognostic systems. (a) OS in IPSS. (b) OS in WPSS. (c) OS in IPSS-R. (d) LFS in IPSS. (e) LFS in WPSS. (f) LFS in IPSS-R. (g) LFS for IPSS-M LR and HR groups. HR includes IPSS-M moderate high, high, and very high groups, while LR includes IPSS-M moderate low, low, and very low groups. The P-values are from the log-rank test. OS, overall survival; LFS, leukemia-free survival; IPSS, International Prognostic Scoring System; WPSS, World Health Organization-classification-based Prognostic Scoring System; IPSS-R, Revised International Prognostic Scoring System; IPSS-M, Molecular International Prognostic Scoring System; LR, lower risk; HR, higher risk.

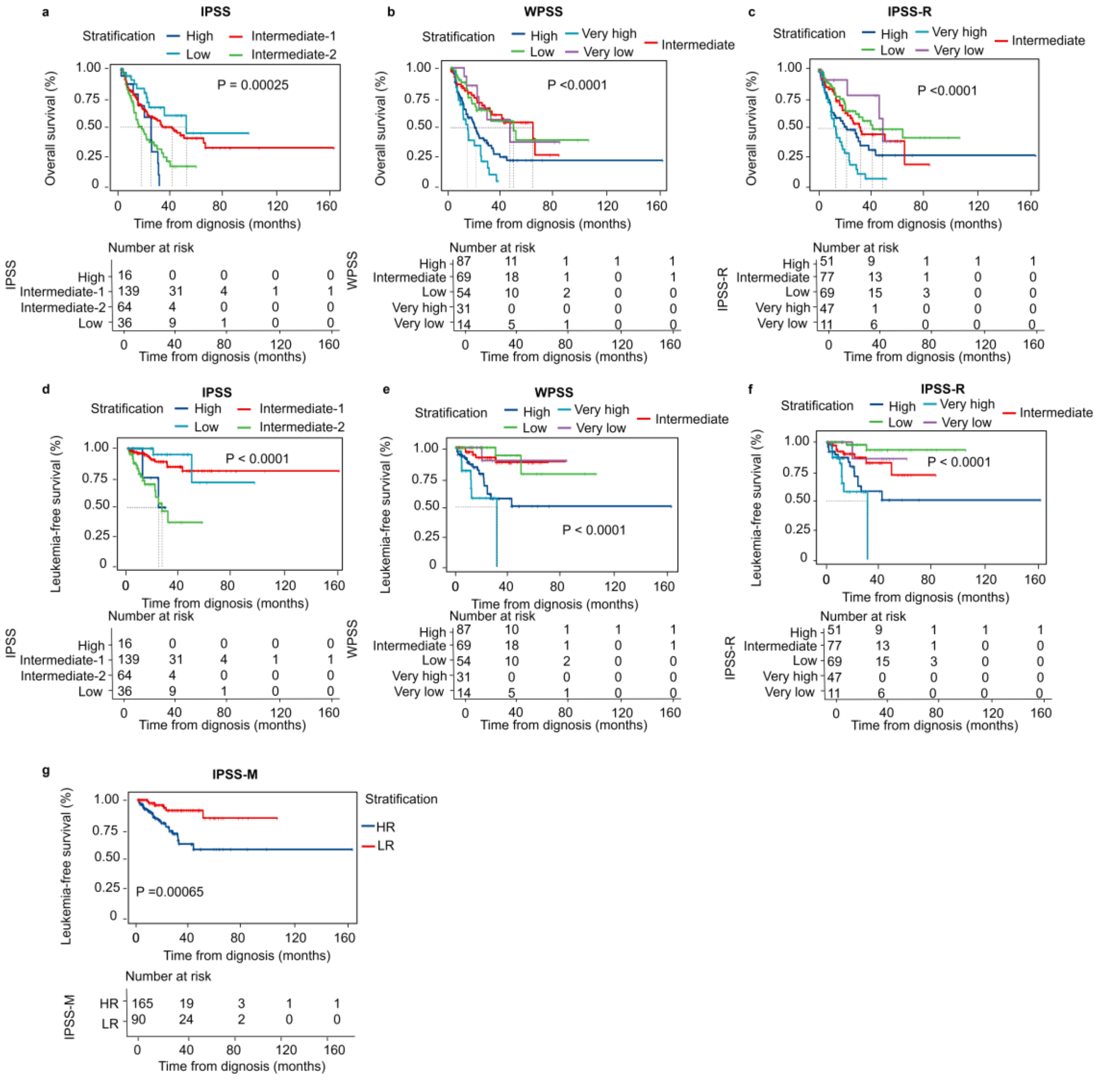

Figure S3 Kaplan-Meier probability estimates of survival outcome per IPSS-M category within each IPSS-R category. (a) Kaplan-Meier probability estimates of OS per IPSS-M category within each IPSS-R category. (b) Kaplan-Meier probability estimates of LFS per IPSS-M category within each IPSS-R category. The P-values are from the log-rank test. OS, overall survival; LFS, leukemia-free survival; IPSS-R, Revised International Prognostic Scoring System; IPSS-M, Molecular International Prognostic Scoring System.

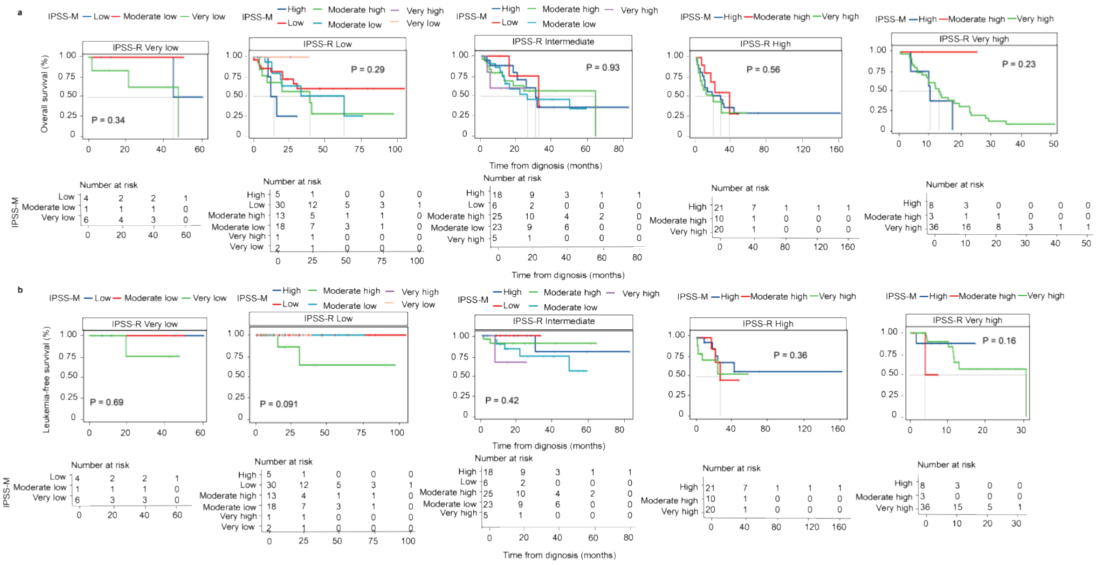

Figure S4 Kaplan-Meier probability estimates of OS for patients who were treated with different therapy per IPSS-M category. The P-value is from the log-rank test. OS, overall survival; IPSS-M, Molecular International Prognostic Scoring System.

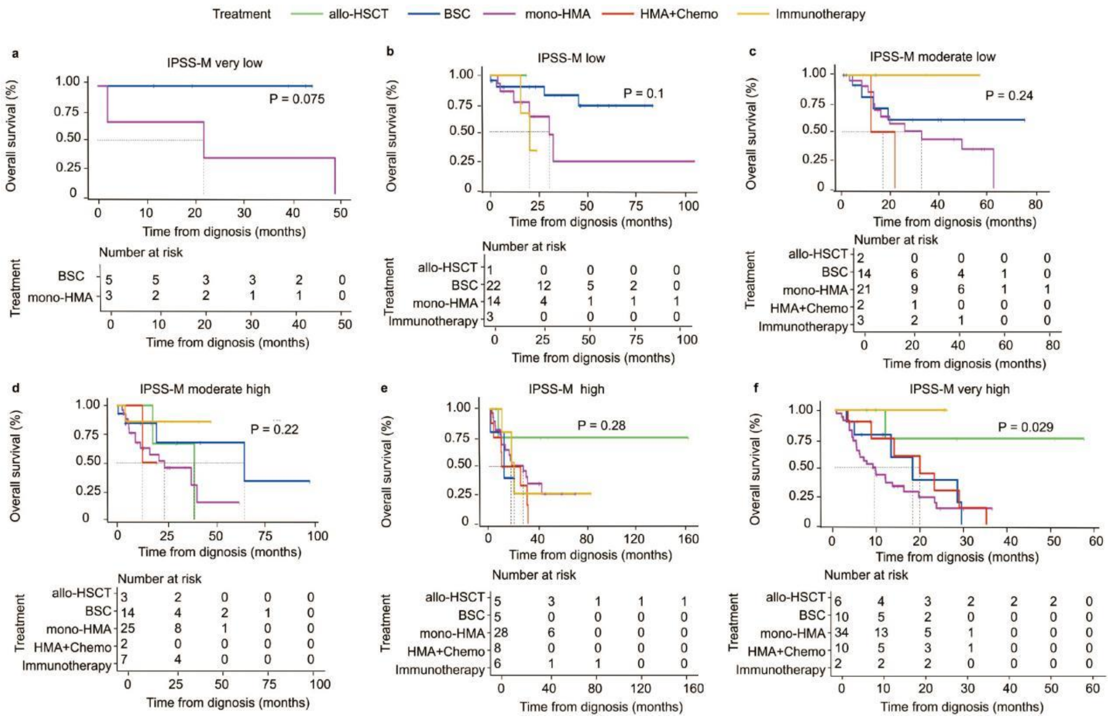

Figure S5 Kaplan-Meier probability estimates of OS for different treatment options in patients with MDS. OS, overall survival; allo-HSCT, allogeneic hematopoietic stem cell transplantation; BSC, best supportive care; mono-HMA, hypomethylation agent monotherapy; HMA+Chemo, HMA combined with chemotherapy.

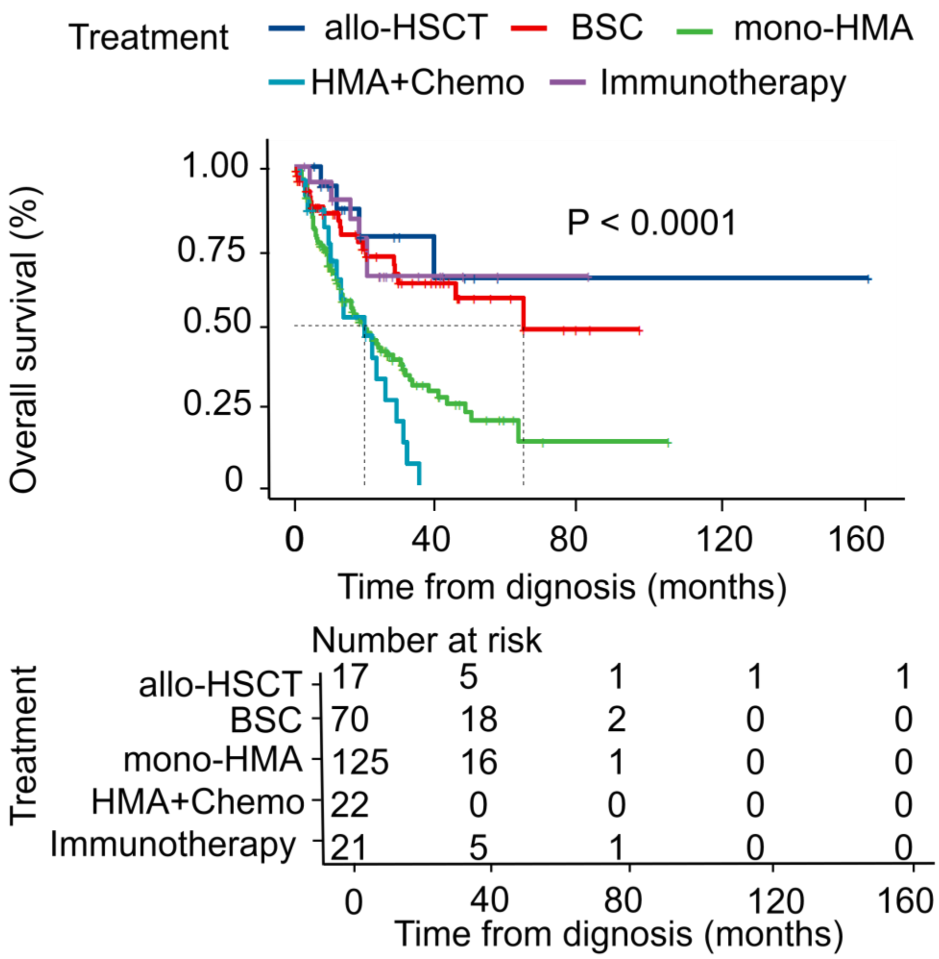

Figure S6 Kaplan-Meier probability estimates of OS in different groups stratified by IPSS-M and IPSS-R who received the same therapy. (a) IPSS-M treated with BSC or immunotherapy. (b) IPSS-M treated with HMAs. (c)IPSS-M treated with transplant . (d) IPSS-R treated with BSC. (e) IPSS-R treated with HMAs. (f) IPSS-R treated with transplant. P-values are from the log-rank test. OS, overall survival; IPSS-M, Molecular International Prognostic Scoring System; IPSS-R, Revised International Prognostic Scoring System; HMAs, hypomethylation agents; BSC, best supportive care.

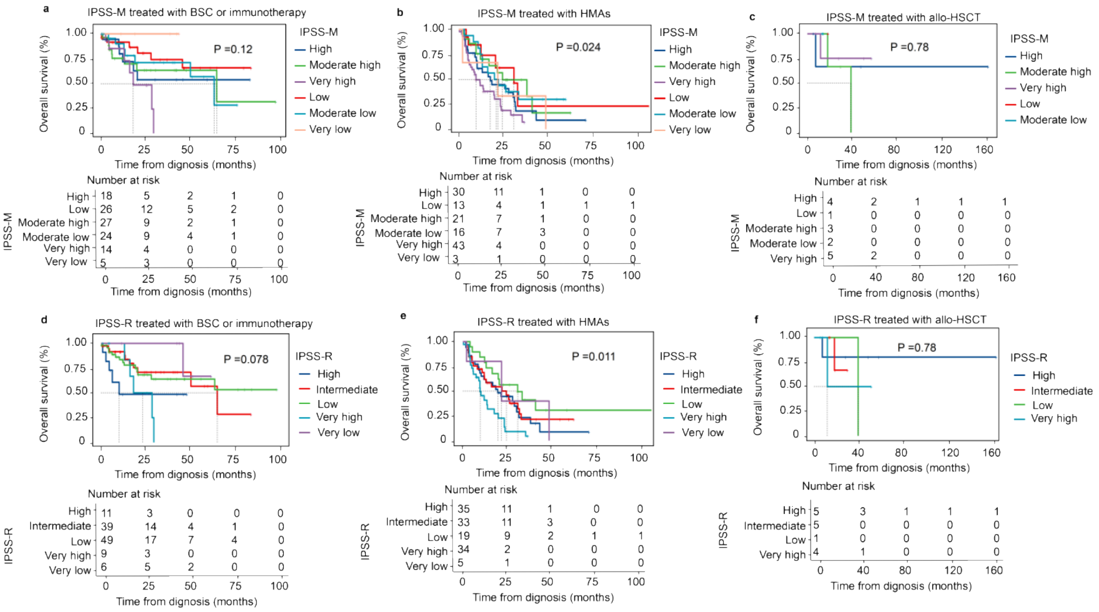

Supplement: Supplementary file 1 [file bs9-5-187-s001.pdf]
